# Interfacial Contact is Required for Metal-Assisted Plasma Etching of Silicon

**DOI:** 10.1002/admi.201800836

**Published:** 2018-10-21

**Authors:** Julia B. Sun, Benjamin D. Almquist

**Affiliations:** Department of Bioengineering, Imperial College London, London SW7 2AZ, UK

**Keywords:** MACE, metal assisted etching, nanofabrication, reactive ion etching, silicon processing

## Abstract

For decades, fabrication of semiconductor devices has utilized well-established etching techniques to create complex nanostructures in silicon. The most common dry process is reactive ion etching which fabricates nanostructures through the selective removal of unmasked silicon. Generalized enhancements of etching have been reported with mask-enhanced etching with Al, Cr, Cu, and Ag masks, but there is a lack of reports exploring the ability of metallic films to catalytically enhance the local etching of silicon in plasmas. Here, metal-assisted plasma etching (MAPE) is performed using patterned nanometers-thick gold films to catalyze the etching of silicon in an SF_6_/O_2_ mixed plasma, selectively increasing the rate of etching by over 1000%. The catalytic enhancement of etching requires direct Si-metal interfacial contact, similar to metal-assisted chemical etching (MACE), but is different in terms of the etching mechanism. The mechanism of MAPE is explored by characterizing the degree of enhancement as a function of Au catalyst configuration and relative oxygen feed concentration, along with the catalytic activities of other common MACE metals including Ag, Pt, and Cu.

## 1 Introduction

Silicon (Si) has long been the cornerstone material in the modern semiconductor industry, establishing a wealth of fabrication techniques in Si nanoprocessing. The impressive diversity in fabricated Si nanostructures is galvanizing the expansion of Si to wide-ranging industries from photovoltaics to biotechnology. Recent applications of Si nanostructures include photonics,[1] solar energy conversion,[2] thermoelectric conversion,[3] energy storage,[4,5] catalysis,[6–8] chemical and biochemical sensing,[9–11] drug delivery,[12] and biological imaging.[13] These diverse applications have driven the development and optimization of a variety of methods for controlling the fabrication of Si structures, ranging from the early years of wet chemical etching before the 1970s and addition of metal-assisted chemical etching (MACE) in the 1990s, followed by the development of reactive ion etching (RIE) for dry etching since the mid-1970s.

In RIE, complex Si nanostructures are fabricated by depositing Si substrates with masking layers of photoresist or compatible metals and selectively etching unmasked Si surfaces through synergistic physical and chemical etching interactions created in gas plasmas of SF_6_, CF_4_, or Cl_2_.[14–19] The dry etching environment provides increased control in selectivity, degree of anisotropy, and etch rate to enable the fabrication of high-aspect-ratio nanostructures.[20] Unlike wet chemical etching, dry etching has the advantage of liquid-free processing which mitigates issues such as stiction and can be more readily incorporated into fully automated processes.

Interestingly, some masking metals have demonstrated a generalized enhancement of etching in plasmas. Mask-enhanced etching of Si and SiO_2_ using Al, Cr, Cu, and Ag masks is known in fluorine-containing etch chemistries.[21–24] The increased etch rates for Si with metal masking are attributed to increases in the concentration of fluorine radicals in the plasma surrounding the metal due to the catalytic production of radicals on the surface of the metal mask, driving the formation of SiF_4_ products in the dry etching reaction. In one report, Au placed upstream of the substrate was shown to increase downstream Si etch rate by 3.6 times in a CF_4_/O_2_ plasma, with the increased etch rate attributed to gas phase transport of Au oxides and fluorine radicals to the substrate.[25] In contrast to Au, Ag and Pt did not have a similar effect, despite Ag demonstrating mask-enhanced etching.[23] In other research, Cu deposits on Si enhance the Si etch rate in F_2_ and Cl_2_ by catalyzing fluorination and chlorination reactions of Si, respectively. [26,27]

While these studies demonstrate the ability of catalytic masking metals to enhance etching in a plasma environment, the fabrication capabilities of mask-enhanced etching and upstream catalyst placement are limited to descriptions of generalized enhancements of etch rate. Two recent studies reporting the local enhanced etching of Si underneath Au in CF_4_/O_2_ suggest a possible nanofabrication strategy, with the authors suggesting, in agreement with mask-enhanced etching, that the observed effects are due to an increase in the production of fluorine radicals within the vicinity of the Au.[28,29] However, outside of these limited studies, there is a shortage of studies exploring the details of how metals can locally catalyze Si etching in reactive plasmas, and if it is merely due to an increase in fluorine radicals or potentially operates via a different mechanism that is similar to the analogous MACE method in wet chemical etching.

Here, we use systematically designed nanoparticles to characterize the mechanism that underlies metal-assisted plasma etching (MAPE) and explore the ability of MAPE to enable selective catalytic etching of Si in a liquid-free environment. Nanostructured metal films are patterned onto Si substrates to catalyze the enhanced etching of Si in an SF_6_/O_2_ mixed plasma. We observe etch rates that are over 1000% higher than the expected rate for Si and proceed through an alternative mechanism of enhancement than previously reported for mask-enhanced etching. We demonstrate that MAPE necessarily relies on direct Si contact with the nanometers-thick metal, does not increase the etch rate of proximal Si, and provides an increase of the etch rate on par with that of MACE. Furthermore, this effect differs from enhanced etching at mask edges created by enhancement of the local electric field. In turn, MAPE is an intriguing fabrication technique for liquid-free metal-assisted etching of silicon nanostructures.

## 2 Results and Discussion

### 2.1 Metal-Assisted Plasma Etching

**Figure 1:**
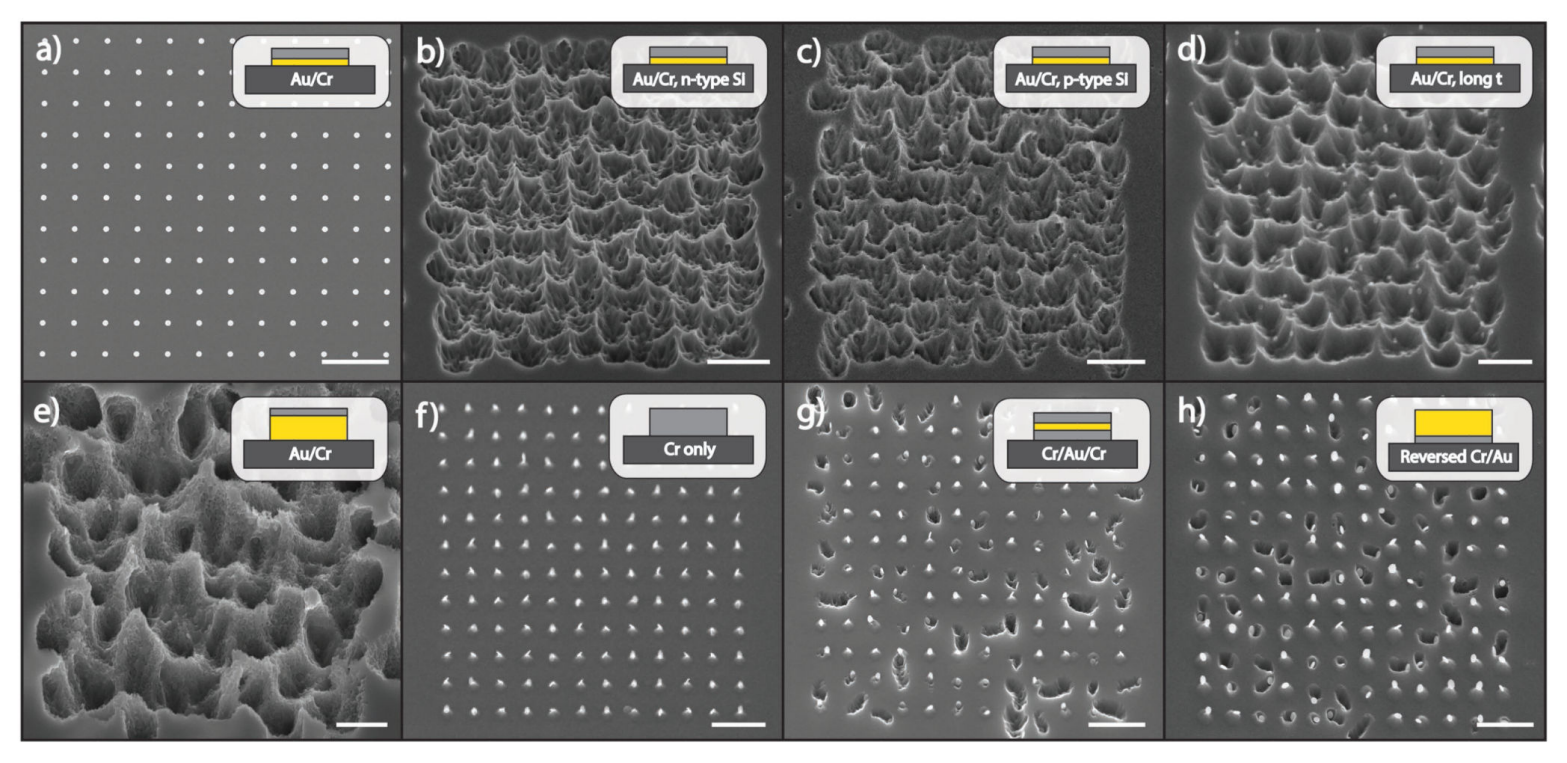
Metal-assisted plasma etching of Si. a) Thin films of 5 nm Au and 10 nm Cr were patterned by electron beam lithography onto Si substrates in 12 × 12 circular nanostructured arrays. Each nanostructure measured 200 nm in diameter. All scale bars represent 2 μm. b) n-type Si substrates patterned with Au/Cr nanostructures were treated for 3 min in a mixed SF_6_/O_2_ plasma at 25% O_2_ concentration. Etch pits characteristic of localized catalytic etching formed underneath each nanostructure while exposed Si at the edges of the array did not exhibit catalytic etching. c) p-type Si substrates patterned and treated at the same conditions produced no significant differences in etching when compared to the n-type Si substrates. d) Si substrates patterned with Au/Cr exhibited similar enhanced etch profiles after etching for 6 min. e) A thicker film of 25 nm Au was patterned onto Si substrates with 10 nm Cr on top. After plasma treatment, etched substrates presented larger etch enhancements with more pronounced etch pits due to the increased Au film. f) Circular Cr-only (25 nm) nanostructures were patterned on Si substrates and subsequently treated in SF_6_/O_2_ for 3 min. Si pillars formed underneath each Cr masking nanostructure and exhibited no evidence of enhanced etching with the removal of the catalytic Au layer. g) Sandwich architecture nanostructures were created by depositing 5 nm Au in between 10 nm layers of Cr. Si substrates with this architecture showed a notable decrease in catalytic etching. Enhanced etch pits occurred only where nanostructures tilted in an orientation to allow the middle Au layer to contact the Si substrate. h) A reverse architecture of 10 nm Cr and 25 nm Au was patterned onto Si substrates. After plasma treatment, a similar catalytic inhibition occurs with Cr serving as an etch stop only when the orientation of the particle results in isolation of the Au from the Si.

To explore the process of MAPE in SF_6_-based plasmas, nano- and microstructure arrays were patterned using electron beam and phase shift lithography on n-type and p-type (100) silicon substrates with resistivity 1–10 Ω cm. SF_6_ was chosen because CF_4_-based plasmas promote fluorocarbon polymerization and deposition that may interfere with the etching behavior. In contrast, SF_6_-based plasmas are restricted to simply etching Si, allowing straightforward observation of the interaction between Si and the metal films. Thin films of Au (5 nm) were deposited using electron beam metal evaporation. A thin film of Cr (10 nm) was deposited on top of the Au film to protect Au from physical etching, creating bilayered metallic nanostructures (Figure 1a). Although Au is known to be incompatible with complementary metal-oxide-semiconductor (CMOS) processes, Au is an important nanomaterial for its widespread use in areas outside of microprocessing and plays a key role in areas such as bio-nanofabrication and devices.[30–33] Cr was chosen due to its high etch resistance in fluorine-based plasmas.[34] Following solvent lift-off to expose the underlying Si, patterned arrays were subjected to RIE with mixed SF_6_/O_2_ gas chemistries for 3 min.

Under normal conditions, SF_6_-based plasma isotropically etches exposed Si to create Si nanopillars underneath each masking structure.[35] However, when the bilayer Au/Cr catalytic structures are etched, areas of Si covered by the patterned metal exhibit preferential and enhanced etching far exceeding the expected etch rates of Si (Figure 1b and Figure S1, Supporting Information). This enhanced etching gives rise to etch pits at the original location of each nanolayered metallic structure, with the boundaries of the etching extending outside those of the original metal nanoparticle but confined locally to the array of the 12 × 12 metal dots (Figure S2, Supporting Information). This enhanced etching is seen in both p-type and n-type silicon, suggesting that the etch enhancement is intrinsic to the Si and not dependent on the type of substrate doping (Figure 1c). Au-only nanostructures lacking the protective Cr capping layer display similar etching; however, the Au-only particles are fully etched away as a result of the direct exposure to the etch plasma, preventing the imaging of the particles following etching (Figure S3, Supporting Information).

To further understand what underpins the enhancement in etching, the length of plasma treatment and metal film thickness were varied in the etch experiments. Interestingly, Si substrates patterned with the same bilayer metal structure but etched for twice as long (6 min) in plasma exhibit no noticeable difference to those etched for 3 min (Figure 1d). At these testing conditions, the etch enhancement does not appear to increase with increasing time. Furthermore, nanostructures with a thicker Au film (25 nm Au/10 nm Cr) produce pronounced etch features in the Si that are deeper and rougher than thinner Au films (Figure 1e). The amount of enhanced etching increases with the increased thickness of Au, suggesting that Au is the main catalytic reagent enabling the metal-enhanced etching of Si, and during the process of RIE the Au is consumed. Taken together, the extent of etching appears to depend primarily on the amount of Au, with higher amounts of Au enhancing etching for a longer time before being consumed. Cr-only nanostructures do not display this enhanced etching, supporting the critical role of Au in catalyzing the MAPE process (Figure 1f).

To determine whether direct Au–Si contact is necessary to facilitate MAPE, we constructed two different configurations of metal nanofilms. First, a thin Au layer was deposited between two layers of Cr to create a sandwich architecture (10 nm Cr/5 nm Au/10 nm Cr), isolating the catalytic layer and preventing its contact with the underlying Si. The etched Si substrates exhibited a notable decrease in MAPE with many Si areas forming pillars underneath the nanostructures, although some regions still form the unmistakable cavities from enhanced etching (Figure 1g). Examination of the nanostructures that maintained the enhanced etch profile suggests that Au–Si contact is required since these nanostructures became oriented in a way that allowed the exposed edge of the sandwiched Au layer to contact the Si surface (Figures S1 and S4, Supporting Information). To further test this concept, a second film configuration was made by placing a thick Au layer (25 nm) above a Cr film (10 nm). In agreement with the sandwich structure, this reverse configuration led to a significant reduction in MAPE, with cases of enhanced etching again occurring only where the nanoparticles appear to reorient themselves so that the top Au layer contacts the Si (Figure 1h and Figure S4, Supporting Information).

These data suggest that Au is unable to diffuse through the Cr layer to the underlying Si to facilitate etching. Furthermore, there is little evidence that etched Au or fluorine radicals produced on the surface are transported through the plasma or diffuse on top of Cr; the pits formed by enhanced etching localize only to the areas patterned with nanostructures following reorientation that places Au in direct contact with Si (Figure 1g,h). If transport of Au or fluorine radicals in the plasma occurs, enhanced etching should be visible in the proximity of the nanoparticles without direct contact between the Au and Si. However, these experiments find that no enhanced etching is observed even within nanometers of Au particles unless there is direct Au–Si interfacial contact. Finally, it is interesting to note that the extent of etching is similar between the sandwich structure (Figure 1g) and inverse structure (Figure 1h), despite the inverse structure having an exposed Au surface area that is over 10× larger than the sandwich structure. This lack of dependence on surface area further supports the concept that the surface of the Au does not generate diffusible products that facilitate the increase in etch rate, and that direct contact between the Au and Si is necessary for enhancing the rate of etching.

The importance of Au–Si interfacial contact is a key feature in the mechanism of the analogous wet etching method MACE. In MACE, Si substrates are deposited with catalytic layers of noble metals such as Au, Ag, Pt, and Pd[36–45] and immersed in an aqueous etchant of hydrofluoric acid (HF) and an oxidative agent such as hydrogen peroxide (H_2_O_2_). Si covered by the noble metal catalyst etches significantly faster than uncovered Si, transferring the pattern of the deposited metal catalyst to the underlying Si. MACE proceeds through electrochemical and mass transport reactions predicated on the reduction of H_2_O_2_ at the metal surface and extraction of electrons from underlying Si, thus injecting holes, at the Si–metal interface to create electron-poor depletion regions in Si that are more susceptible to etching by HF.[46–51]

From this data, several conclusions are possible regarding the mechanism of MAPE. First, Au acts as a catalytic reagent to promote the enhanced etching of Si in SF_6_/O_2_ plasma. The enhanced etching effect is far more pronounced than typical Si etching. Second, direct interfacial contact of Au on Si is critical for the catalytic etching reaction to proceed. The enhanced etching can be blocked by entirely isolating the Au layer from the Si surface with a nonreactive metal like Cr. Third, the degree of enhancement depends on the amount of catalytic Au present, suggesting that Au is consumed during the process of MAPE via secondary mechanisms that are present in addition to Si etching; Au may diffuse into the Si, undergo a chemical reaction that likely creates gold oxides and fluorides, or be sputtered and etched away by the high-energy plasma. In these experiments, the amount of remaining Au measured on the Si substrates following etching was less than the detection limit for energy-dispersive X-ray spectroscopy. If the etch is well controlled, it appears that MAPE has the potential to operate as a self-limiting etch that terminates after depletion of the Au catalyst. This aspect is different from MACE where the catalytic metal remains on the etched substrate and the etch process ends by manual removal of the substrate or neutralization of the chemical etchants. Note that although it appears that MAPE consumes the Au, it is referenced here as a metal catalyst because we hypothesize that the consumption of the Au is likely due to the aforementioned secondary reactions and not as part of the Si etching reaction, positioning Au as a catalyst in our proposed mechanism underlying MAPE. While the formation of volatile Au–Si compounds are technically possible, this is unlikely to underpin the enhanced etching due to preferential formation of SiF_4_, Au–Si generally requiring specialized routes of synthesis,[52] and the lack of volatile Au–Si compounds depleting Au catalysts at the low pressures and elevated temperatures found during vapor–liquid–solid growth of Si nanowires.

Finally, transport of the plasma ions to the Au–Si interface is required. Data from Si substrates patterned with large lateral Au/Cr bilayer structures of the same thickness (5 nm Au/10 nm Cr) and etched in SF_6_/O_2_ further supports this requirement (Figure S5, Supporting Information). Si substrates exhibit similar enhanced etching to the nanopatterned substrates, but only at the Au–Si–plasma interfaces. Etching appears to proceed from the perimeter of the larger structures suggesting inward diffusion from the interface. Furthermore, images from early etching with nanoparticles suggest that pillars created via conventional SF_6_/O_2_ plasma etching precede metal-enhanced etching (Figure S6, Supporting Information), with enhanced etching likely starting following exposure of the Au–Si interface to the plasma. Importantly, these data support a conclusion that metal nano- and microstructures catalyze an enhanced etching phenomenon that is divergent from all previously reported plasma-enhanced edge effects and mask-enhanced etching. Catalytic activity is not purely limited to the mask edges from a concentration of reactive species in the vicinity of the mask or local enhancement of the field strength. Instead, the Au in direct contact with Si actively promotes enhanced etching in SF_6_/O_2_ plasmas.

### 2.2 Effects of Oxygen Content on MAPE

**Figure 2:**
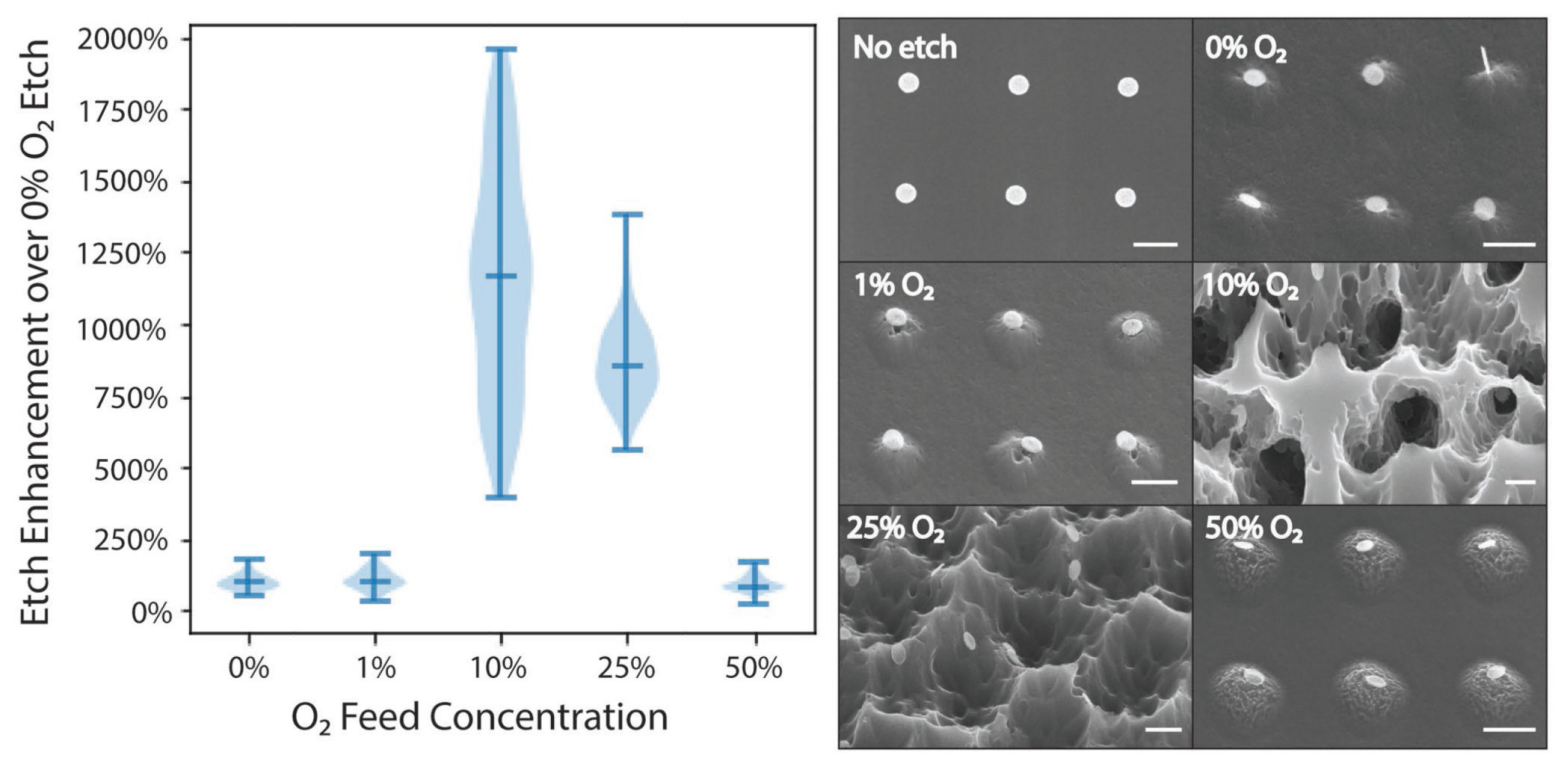
Effect of O_2_ concentration on MAPE. Si substrates were patterned with 5 nm Au/10 nm Cr and etched for 3 min in SF_6_/O_2_. By maintaining the total flow rate at 40 sccm and manipulating the O_2_ flow rate, we examined the effect of O_2_ concentration on the etch enhancement over a 0% O_2_ etch. (left) Violin plots indicating the median and range for each condition. Little or no etch enhancement was measured at 0% and 1% O_2_ while treatment in 10% O_2_ exhibited a maximal amount of catalytic enhancement. A smaller degree of etch enhancement occurs at 25% O_2_ with the disappearance of etch enhancement at 50% O_2_. (right) Representative images of etching. All scale bars are 400 nm.

Having established that SF_6_-based plasmas facilitate MAPE, we then aimed to determine the impact of oxygen in the process. The addition of O_2_ to SF_6_ is known to increase the F-atom concentration, increasing overall Si etch rate by preventing the recombination of F atoms with SF_x_ radicals through the formation of SO_2_ and SOF_4_.[20] To determine if MAPE maintains this O_2_ dependence, we evaluated a range of O2 concentrations from 0% to 50% at a constant total flow rate and pressure (Figure 2; Figure S7, Supporting Information). Minimal or no MAPE occurs when the fraction of O_2_ is 1% or less, whereas at 10% O_2_ we observe the maximum enhancement of the MAPE process. This finding follows a similar trend to previous reports of maximal Si etch rates occurring between 10% and 40% O_2_ composition for both SF_6_/O_2_ and CF_4_/O_2_ etch chemistries,[14,53,54] though the maximum etch rate has been known to vary based on system-specific parameters.[20] The MAPE process is observed through 25% O_2_ but exhibits a significant decrease at 50% O_2_. This decrease is supported by previous observations of an inversion in etch rates above 40% O_2_ due to competitive adsorption of increasing oxygen species onto Si reaction sites over fluorine species.[14] Taken together, these data suggest that similar to its effect on normal Si etch rates for fluorine/oxygen plasmas, the relative concentration of O_2_ in the plasma plays a significant role in determining the etch rate of MAPE.

### 2.3 Translation of Additional MACE Metals to MAPE

**Figure 3:**
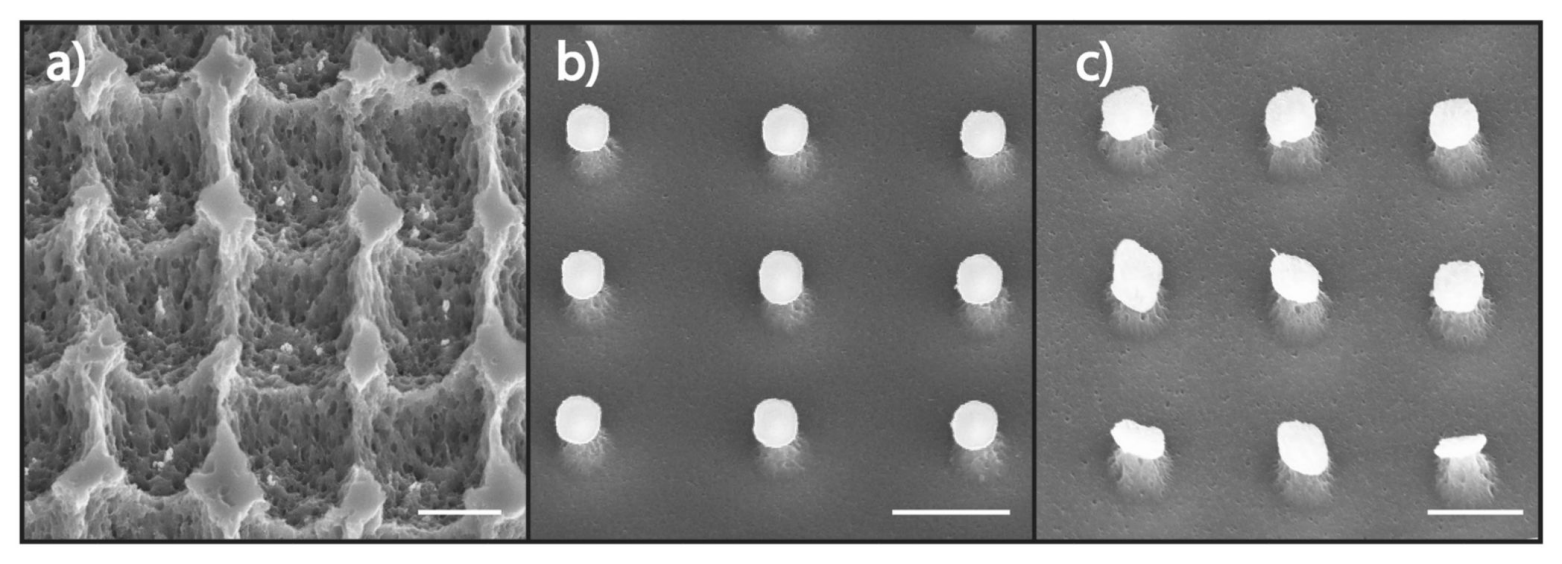
MACE metals in MAPE. a) Microstructure arrays patterned by phase shift lithography on Si substrates. Twenty five nanometers of Ag was deposited onto the substrate to form 1.0 × 1.3 μm rectangular structures. After treatment in an SF_6_/O_2_ plasma for 3 min, Si substrates exhibited etch profiles indicative of MAPE. Little of the Ag microstructures remained in the etch pits, likely due to the low resistance of Ag to etching in SF_6_ plasmas. b) Similar microstructures were created with 25 nm Pt. After treatment, Si pillars formed underneath each Pt microstructure with no evidence of enhanced etching. c) Si substrates patterned with 25 nm Cu also did not exhibit MAPE after plasma treatment although the etch produced increased roughness on the Si surface. All scale bars are 2 μm.

In addition to Au, liquid phase MACE is routinely achieved using other noble metals such as Ag and Pt, and to a lesser degree, Cu. Due to the mechanistic similarities of Au–Si interfacial contact in MACE and MAPE, we sought to determine if the catalytic effect is transferrable to MAPE where the reaction may proceed via similar electrochemical mechanisms. Si substrates were patterned with microstructures of the common MACE catalysts Ag, Pt, and Cu and etched using the same conditions as those in Figure 1. Similar to substrates patterned with Au films (Figure 1; Figure S3, Supporting Information), Si substrates patterned with Ag films exhibit enhanced etching suggesting that Ag has a comparable ability to facilitate MAPE (Figure 3a). However, substrates patterned with Pt do not show increased etching of the Si substrate (Figure 3b) despite Pt MACE being faster than Au MACE.[36] Substrates patterned with Cu also do not exhibit any catalytic etching but instead induce a rougher Si surface following etching than Pt (Figure 3c).

The difference in the catalytic effect between MACE and MAPE suggests that the mechanism of MAPE deviates from that of MACE, although the exact reason why a metal will function as a catalyst in MACE but not MAPE is not currently fully understood. Unlike in MACE, where the reduction potential of the metal catalyst in relation to the Si ionization potential predicts whether a metal will function as a MACE catalyst,[51] reduction potential does not correctly predict the noncatalytic behavior of Pt and Cu in MAPE. Although shown not to correlate with MACE activity, previous studies have demonstrated that changing the work function of metals can enhance their catalytic activity.[55] In agreement with MACE findings, measurements of the work function of catalyst-only and catalyst/Cr configurations do not accurately predict the ability to facilitate MAPE (Figure S8, Supporting Information).

The diffusion of metals into Si substrates and the reverse diffusion of Si into metal is fast even at modest temperatures.[56] Molecular dynamics simulations of the MACE process show that the high electronegativity of Au permits catalytic Au atoms to draw electrons from nearby Si atoms, creating active etching sites in the Si for electron-rich oxidizing agents.[57] Increased diffusion at the metal–Si interface may facilitate enhanced Si etching by increasing the overall mobility of metal ions and their ability to modulate the electron density of Si. This contradicts the mechanism underpinning MACE, where the catalytic metals serve to extract electrons/inject holes and neither the Si nor metal noticeably enriches with diffused atoms. Though the diffusivities of Pt and Cu do not significantly differ from the diffusivities of Au and Ag on Si,[58] the formation of metal oxides on the surface of the catalyst metal when exposed to O_2_ and/or the ability to form stable compounds with Si, as opposed to solid solutions, may provide insight into a possible mechanism. Binary oxides formed from Pt and Cu have higher thermodynamic stability when compared to oxides formed from Au and Ag on Si.[59] Since MAPE occurs in the presence of O_2_, the increased stability of these oxides may negatively impair the diffusion at the metal–Si interface,[60] in turn inhibiting the enhanced etching. Furthermore, Pt/Si and Cu/Si readily form stable compounds,[60,61] potentially reducing their ability to act as Si oxidizing agents.

An alternative mechanism underpinning the enhanced etching is the possible formation of surface-associated metal fluorides. Metal fluoride compounds such as AuF_5_, AgF_2_, and AgF_3_ are known to be extremely strong oxidizing agents[62–64] and may draw electrons from Si to create active sites for etching. These metal fluorides readily form when the metal surface is exposed to fluorine species in plasma[65–67] and may more readily compete with the formation of metal oxides due to increased thermodynamic stability.[68] High electron affinity metal fluorides formed with Pt are possible, but compounds such as PtF_6_ are known to be volatile at room temperature,[69] potentially resulting in rapid removal from the Si–Pt interface. Overall, this mechanism relying on the production of metal fluorides to oxidize underlying Si is similar to the MACE mechanism wherein the metal catalyst serves to oxidize underlying Si for etching. Notably, oxidation of the Si should simply enhance the standard rate of Si etching in fluorine-based plasmas for a given condition. Here, the etch rate dependence for MAPE with O_2_ concentration (Figure 2) mirrors the behavior of standard SF_6_/O_2_ etching, and the minimal etching of SiO_2_ by SF_6_ is also maintained for MAPE (Figure S9, Supporting Information).

The widening of the etching boundaries in the current demonstration of MAPE is predicted to be due to uncontrolled particle movement in the high-energy plasma environment. Localized jumping of particles during etching may cause the etching boundary to extend past that of the metal nanostructures; this is evidenced by the scalloped features formed by contact with the nanoparticles in the widened etch boundaries within the original 12 × 12 array, but not extending longer range outside of the etching area. Within the scalloped areas, particles can be seen that are more randomly arranged than at the start of etching, whereas outside of the arrays there are no particles and no enhanced etching (Figure S2, Supporting Information). Long-range diffusion of metal ion into silicon is also a potential theory; however, this is unlikely since the etch boundary profiles do not correspond to the expected exponential decay profiles in mass diffusion. Going forward, the introduction of mechanisms that limit this particle jumping phenomenon may decrease the observed etch boundary extension and enable more precise patterning via MAPE.

### 2.4 Proposed Mechanism Underpinning the Process of MAPE

**Figure 4:**
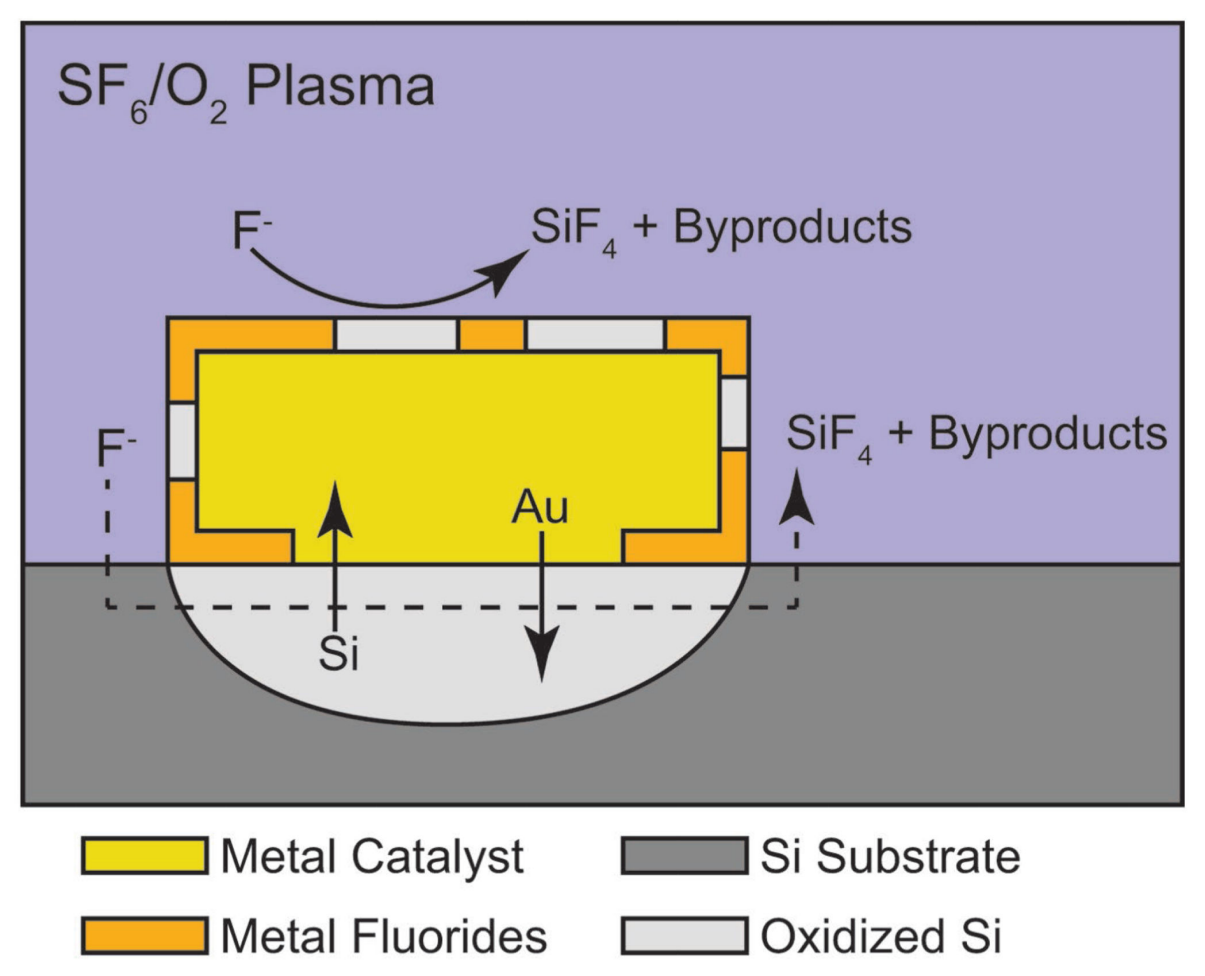
The proposed mechanism of MAPE. Diffusion occurs across the Si–metal interface, resulting in oxidation of both the Si underlying the metal and Si diffusing to the surface of the metal. In the presence of SF_6_/O_2_ plasma, surface-associated metal fluorides form that further oxidize the Si. Fluorine radicals present in the plasma etch the oxidized Si at a faster rate than standard Si.

Based on the data presented here, we propose the following potential MAPE mechanisms (Figure 4): 1) The generation of an SF_6_/O_2_ plasma creates reactive species including F and O radicals, SO_2_F_2_, SOF_4_, and other charged species. 2) Diffusion occurs across the metal–Si interface as metal ions diffuse into the underlying Si and Si atoms diffuse through the metal layer to the surface. 3) Si is oxidized on the surface of the metal or by electron density modulation by dissolved metal ions in the underlying Si. In addition, the surface of the metal chemically reacts with fluorine species to form metal fluoride oxidizing agents. The surface-associated metal fluorides oxidize the underlying Si at the metal–Si interface. Finally, 4) the etching reaction proceeds with the transport of fluorine species to the oxidized Si, the formation of SiF_4_ products, and subsequent removal of byproducts via the plasma. Similar to liquid phase MACE, MAPE likely proceeds through both thermodynamically driven redox and mass transport reactions. However, further investigation is essential to fully understand the underpinning mechanism and establish a predictive model for catalytic metal activity in dry plasmas, along with detailed observations and tracking of the Au to confirm its role as a true catalyst in MAPE.

## 3 Conclusion

In summary, these data have uncovered a previously unreported mechanism by which Au/Ag facilitates enhanced etching of Si in dry SF_6_/O_2_ plasmas. By utilizing patterned metallic nano- and microstructures to systematically study the enhanced etching of Si, these results support an interface-driven mechanism that is fundamentally different than the increased production of fluorine radicals that was suggested in previous reports of enhanced etching by noble metal masks. Instead, this interfacial mechanism is similar, but still divergent from, the interfacial contact necessary for MACE. The MAPE process exhibits a rate dependence on oxygen feed concentration similar to that of classic silicon etching. Interestingly, not all metals that function as MACE catalysts can function as MAPE catalysts, supporting the conclusion that the plasma-based mechanism deviates from that of liquid phase MACE. By further developing MAPE, limitations of liquid phase fabrication such as fluid flow effects, stiction, and solute deposition are eliminated. With further development of the techniques described here, MAPE may provide an exciting strategy for Si processing using nanometers-thick metal catalysts in liquid-free environments.

## 4 Experimental Section

### Patterning of Periodic Arrays

Nanostructures were patterned onto p- and n-type (100) silicon wafers with resistivity 1–10 Ω cm (Pi-Kem, UK) spin-coated with polymethyl methacrylate 950-A2 (Microchem, USA), and patterned using a RAITH 150 TWO electron beam lithography system (Raith GmbH, Germany). Following exposure, patterns were developed in a 1:3 mixture of methyl isobutyl ketone in isopropyl alcohol and treated in oxygen plasma in a Diener plasma generator (Diener electronic GmbH & Co. KG, Germany) to remove excess photoresist scum and produce final circular features with an approximate diameter of 200 nm. Microstructures were patterned onto silicon wafers spin-coated with S1805 photoresist (Microchem, USA) using phase shift lithography. A borosilicate mask etched with lines 1.5 in. in length and 1.5 μm thick with 1.5 μm spacing was used to perform double exposures at 5 mJ cm^−2^ with the mask turned 90° in between exposures on a Quintel Q4000-6 Mask Aligner (Neutronix Quintel, USA). Patterns were developed in MICROPOSIT MF-26A (Dow Electronic Materials, USA) to produce final rectangular patterns measuring approximately 1 × 1.3 μm.

### Thin Film Deposition

After patterning, layers of Au and other metals were deposited in an Edwards A500-FL500 electron beam metal evaporator (Edwards High Vacuum International, UK) to form thin layers ranging from 5 to 25 nm in thickness. Uniformity was confirmed using a DektakXT surface profilometer (Bruker Corporation, USA). Lift-off of excess photoresist and metals was performed using MICROPOSIT Remover 1165 solvent (Dow Electronic Materials, USA).

### RIE

The masked silicon wafers were etched in a parallel plate PlasmaPro NGP90 plasma processing system (Oxford Instruments, UK). RIE was performed at 175 mTorr using a forward power of 50 W, and the total gas flow of SF_6_/O_2_ was held constant at 40 sccm. The total concentration of O_2_ was varied by changing the relative gas flow rates of SF_6_ and O_2_.

### Imaging

Visualization of the etched structures was performed with regular and tilting SEM on a Zeiss XB1540 (Carl Zeiss AG, Germany) and LEO Gemini 1525 FEGSEM (LEO Electron Microscopy Inc, USA). Tilting images were executed at 45° for etched structure imaging and 90° for side fractural imaging.

### Etch Enhancement Measurements

Si substrates following plasma treatment were fractured for cross-sectional imaging of the etch pits. The vertical etch depth was measured at the center of the Si pillars or catalytically etched pits using ImageJ. Ten measurements were made for every 12 × 12 nanostructured array. The etch enhancement over 0% O_2_ was measured by normalizing each measured value to the mean value for the Si substrate treated at 0% O_2_. For substrates that formed etch pits, the mean value at 0% O_2_ was added to the vertical etch measurement to account for the pitting effect.

## Supplementary Material

Supporting Information is available from the Wiley Online Library or from the author.

Supplemental Data
