# Supplementary material for: Interfacial Contact is Required for Metal-Assisted Plasma Etching of Silicon
Source: Adv Mater Interfaces. Author manuscript; Available in PMC 2019 Jan 2. (PMC6314446; doi:10.1002/admi.201800836)
Supplement: Supplemental Data [file NIHMS80257-supplement-Supplemental_Data.pdf]

Supporting Information for:

Interfacial Contact is Required for Metal-Assisted Plasma Etching of Silicon

Julia B. Sun and Benjamin D. Almquist
Department of Bioengineering, Imperial College London

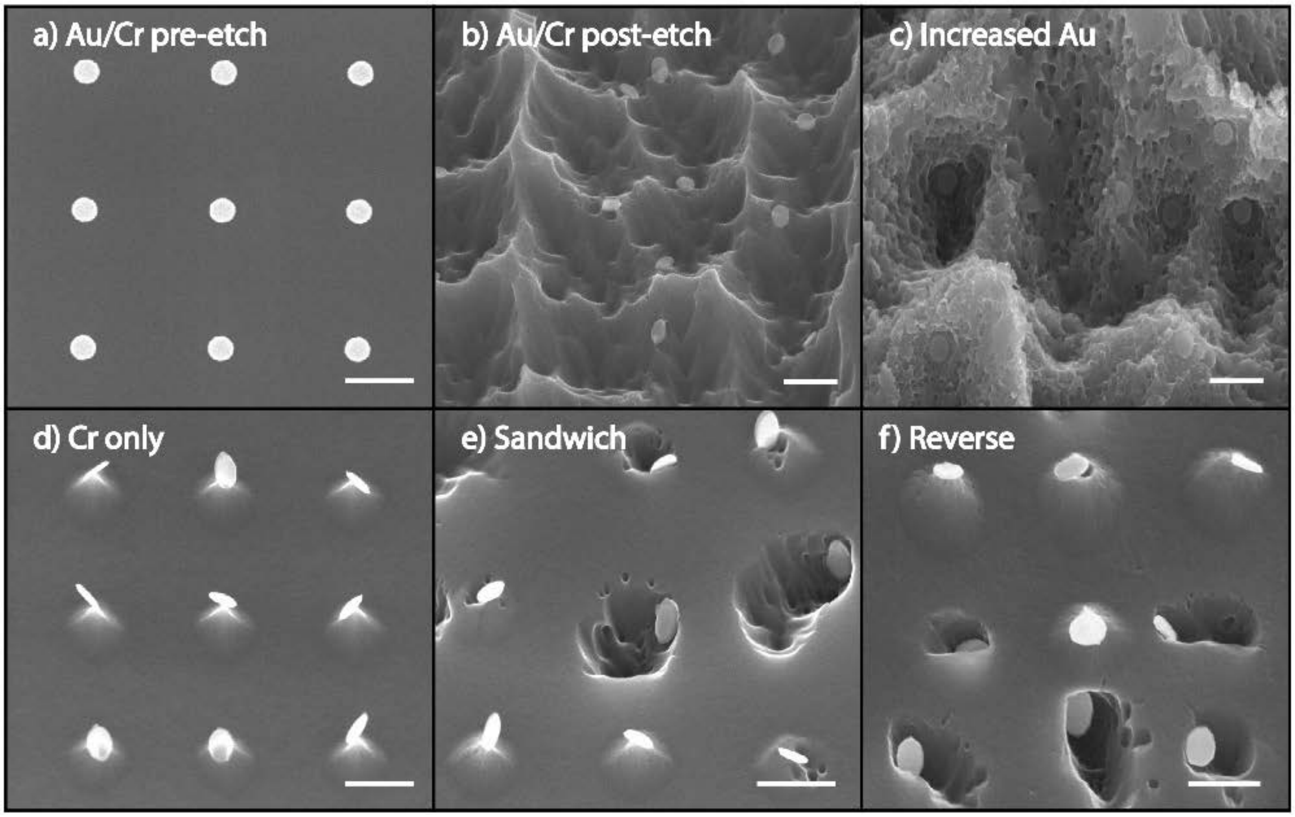

Figure S1. Enlarged images of MAPE. a) Si substrates were patterned with circular nanostructures by electron beam lithography and deposited with 5 nm Au/10 nm Cr. All scale bars represent 500 nm. b) Si substrates were etched for 3 minutes in SF_6/O_2 at 25% O_2 concentration. Au/Cr nanostructures catalyzed enhanced etching of the underlying Si substrate and remained visible on the walls of the etch trenches. c) Nanostructures with a thicker Au layer (25 nm Au/10 nm Cr) caused an enhanced MAPE effect creating a rougher etched surface and deeper etch trenches. d) Cr only (25 nm) nanostructures formed traditional pillars in the underlying Si after etching at the same conditions. The pillars formed underneath each Cr nanostructure, which acted as circular etch masks. e) Sandwich architecture nanostructures made of 10 nm Cr/5 nm Au/10 nm Cr impeded MAPE to form Si pillars. However, enhanced etching was observed wherever the sandwich nanostructures tilted to allow direct contact of the nanostructure edge and Si surface. f) Reverse architecture nanostructures made of 10 nm Cr and 25 nm Au on top also impeded MAPE to form Si pillars. Similarity, tilted nanostructures that enabled contact between the upper Au layer and Si surface exhibited the enhanced etch trenches of MAPE.

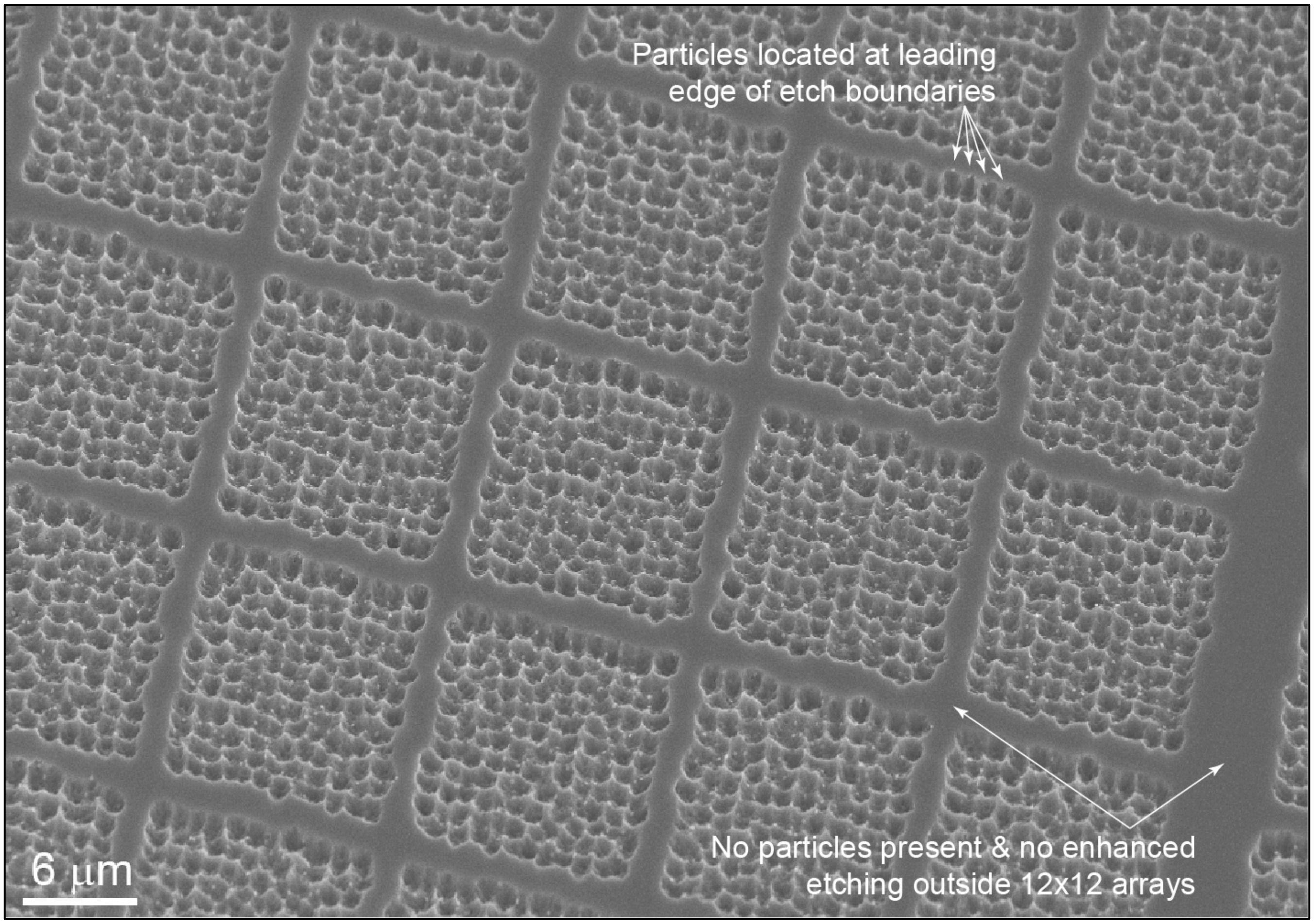

Figure S2. Etching is limited to local regions within the original 12x12 nanoparticle array. No particles and no etching are observed in areas between the arrays, suggesting the stochastic movement of particles is limited to the local areas within the array instead of long-range movement. Nanoparticle are also seen at the leading edge of etching boundaries, further supporting the necessity of interfacial contact between Au and Si in catalyzing the process of MAPE. Nanoparticles are 5 nm Au/10 nm Cr, similar to those in Figure 2a-d and Figure S5.

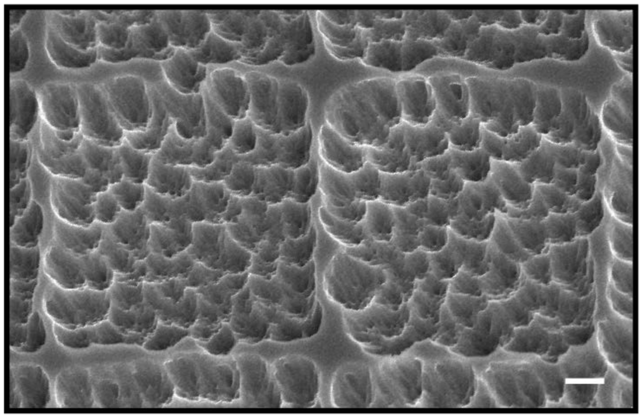

Figure S3. Metal-assisted etching with pure Au nanoparticles. 25 nm-thick Au nanoparticles were patterned onto a Si substrate by electron beam lithography and subsequently etched for 3 min in SF_6/O_2 at 25% O_2 concentration. The Au nanoparticles enhanced etching of the Si substrate, but no particles remain after etching due to the low etch resistance of Au. The scale bar represents 2 μm.

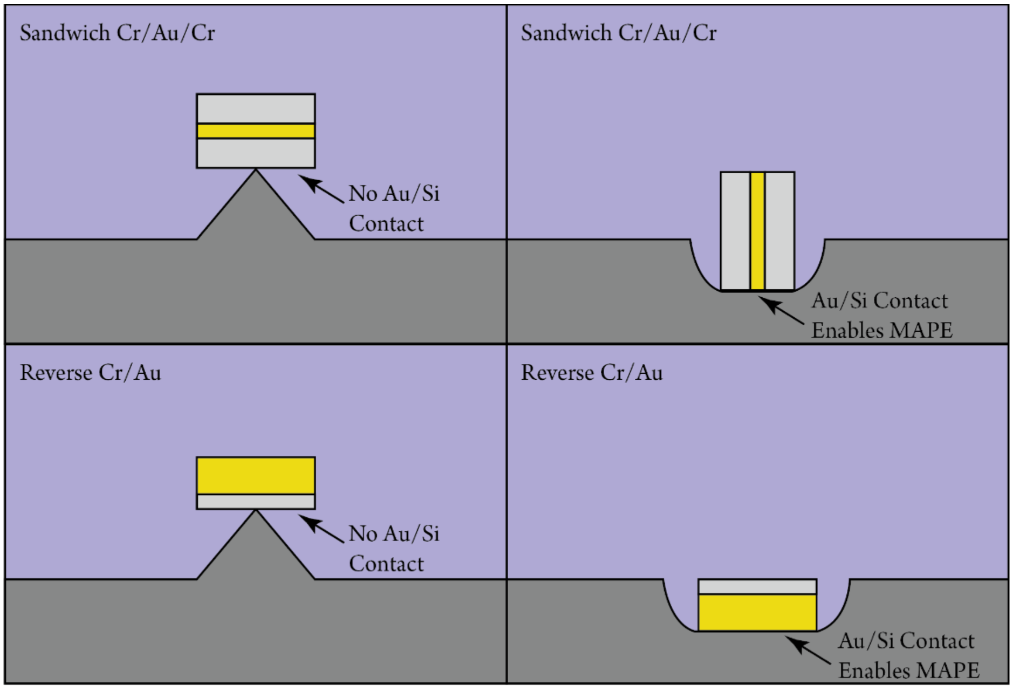

Figure S4. Schematic of sandwich and reverse catalyst architectures during MAPE. In the sandwich architecture (top panels), contact between the catalytic Au layer and Si surface is inhibited by the surrounding Cr layers. This allows the sandwich nanostructure to act as an etch mask to form Si pillars during SF_6/O_2 treatment. When the nanostructure is tilted to allow the middle layer to contact the Si surface, the Si undergoes enhanced etching to form a trench underneath the nanostructure. In the reverse architecture (bottom panels), the catalytic Au layer is prevented from contacting the Si surface with a thin layer of Cr. This structure effectively inhibits MAPE and forms a Si pillar during etching. When the nanostructure is shifted to allow the Au layer to directly contact Si, MAPE proceeds and creates a deep etch trench at the contact interface.

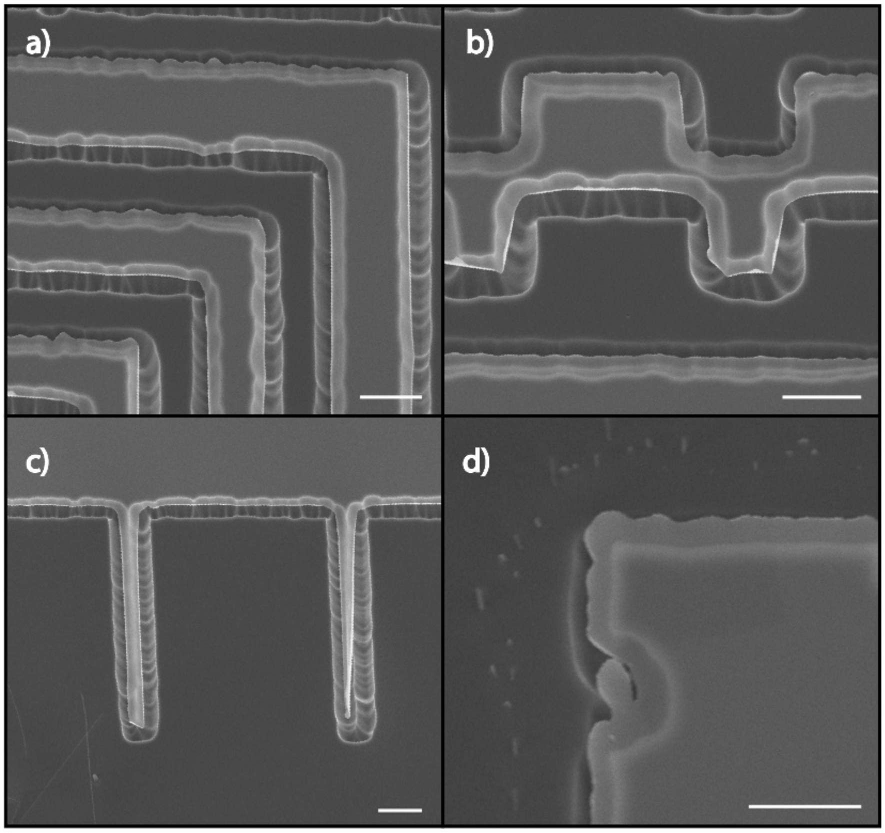

Figure S5. Enhanced etching in large lateral structures. Large lateral structures were patterned onto Si using UV lithography and deposited with 5 nm Au/10 nm Cr. Si substrates were etched for 3 min in SF_6/O_2 at 10% O_2 concentration and exhibited MAPE only at the Au/Si/plasma interfaces. Scale bars for panels a-c represent 10 μm and the scale bar for panel d represents 5 μm.

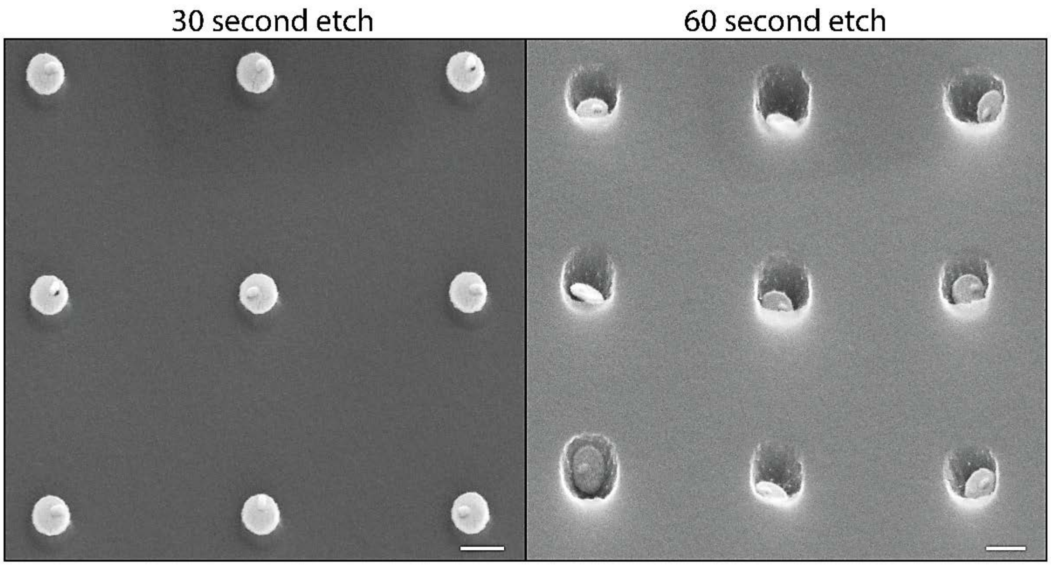

Figure S6. Early Stages of Etching. (left) At early stages of etching, pillars form underneath Au/Cr nanoparticles, similar to standard SF_6/O_2 etching. (right) At longer times, enhanced etching due to Au-Si contact dominates, suggesting that exposure of the Au-Si interface to the plasma is necessary for enhanced etching to occur. Nanoparticles are 5 nm Au/10 nm Cr, similar to those in Figure 2a-d. Scale bars represent 200 nm.

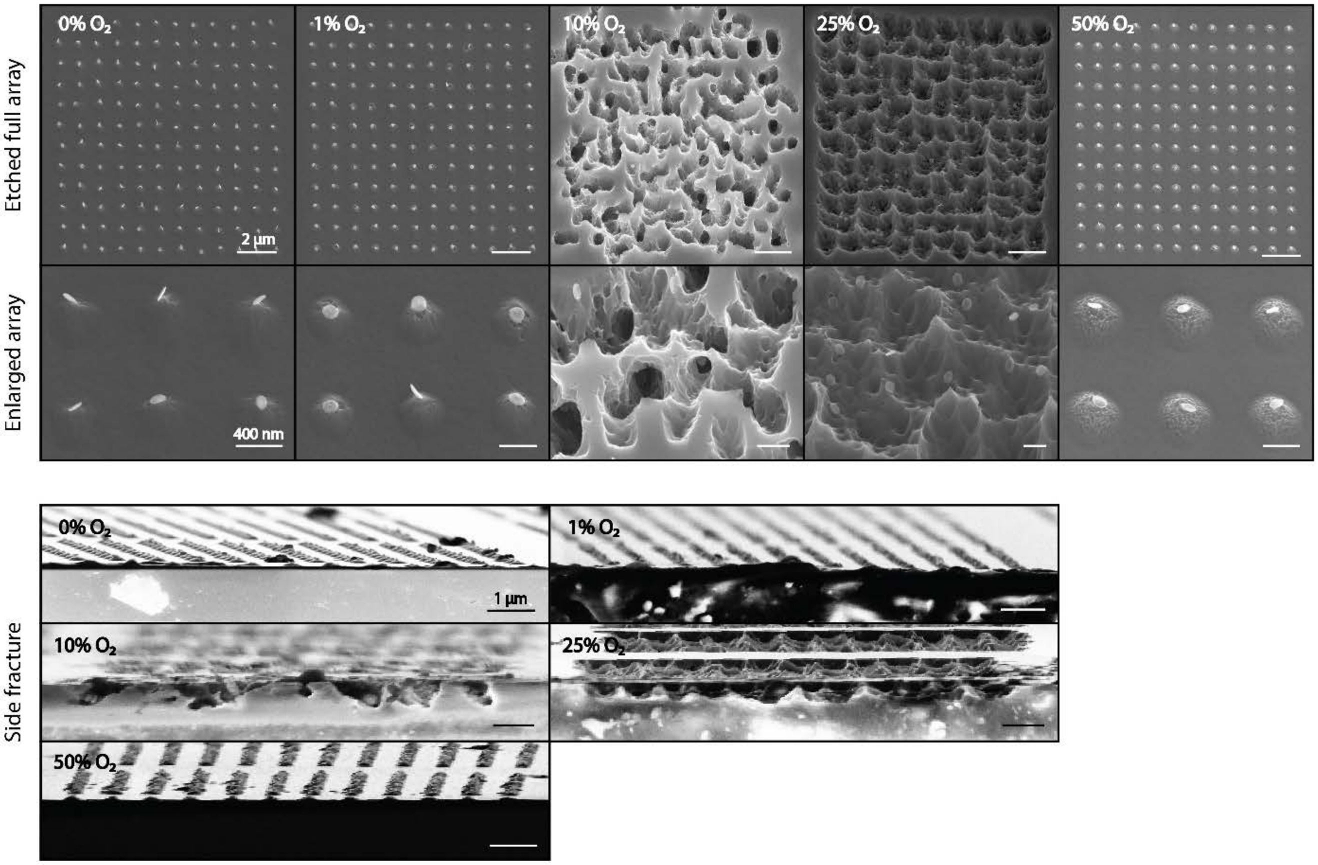

Figure S7. Enlarged images of the effect of O_2 on MAPE. Si substrates patterned with 12x12 nanostructure arrays were etched in SF_6/O_2 for 3 minutes at various O_2 feed concentrations. The full nanostructure arrays and enlarged arrays after etching are depicted in the top panels. All scale bars for the full arrays represent 2 μm and all scale bars for the enlarged arrays represent 400 nm. Enhanced MAPE was observed for all substrates etched at 1%, 10% and 25% O_2 concentrations with maximal enhancement occurring at 10% O_2 concentration. For etch enhancement measurements, Si substrates were fractured and imaged at 90°. The bottom panels depict the cross-sectional fractures for the same five etch conditions with all scale bars representing 1 μm. Ten vertical depth measurements were made at the centers of the Si pillars or etched pits for every array. Measurements were normalized by the mean measurement for Si substrate treated at 0% O_2 to obtain the etch enhancement over 0% O_2.

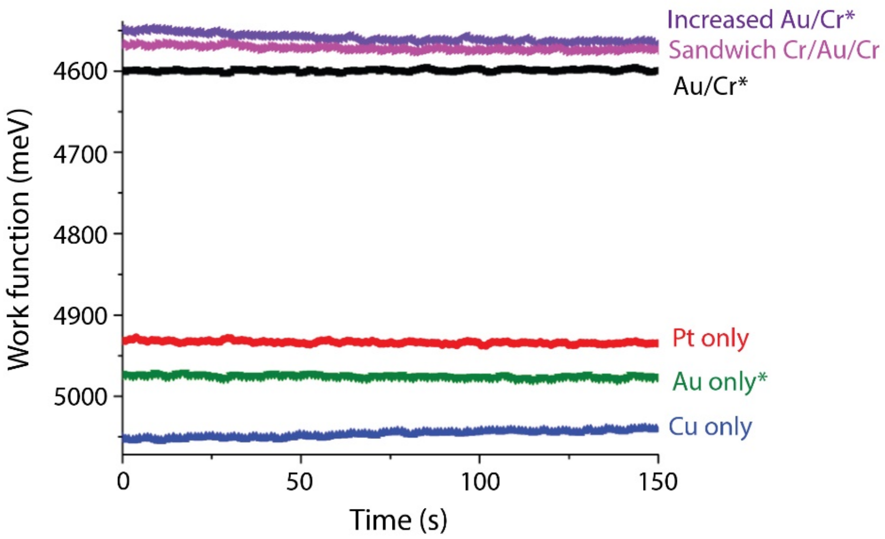

Figure S8. Work function measurement of catalyst architectures by Kelvin Probe. The work function of uniform metallic layers for six catalyst architectures were measured by kelvin probe. The catalyst architectures that performed MAPE in SF_6/O_2 are denoted by an asterisk (*). Work function does not accurately predict catalyst function in MAPE.

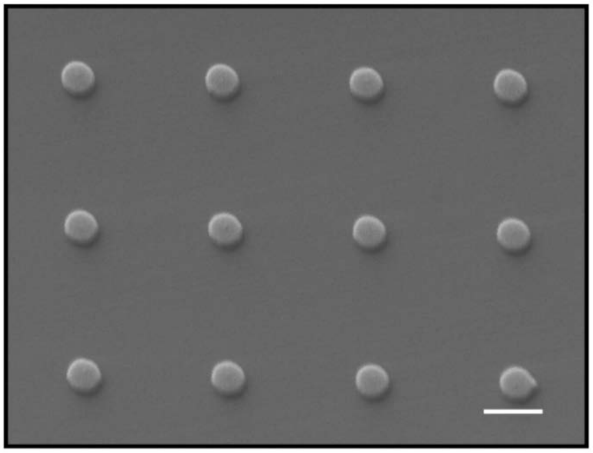

Figure S9. Inhibition of metal-assisted etching in oxides layers. 5 nm Au/10 nm Cr nanoparticles were patterned onto a Si substrate with a 500 nm-thick thermally grown SiO_2 top layer. The SiO_2/Si substrate was etched for 3 min in SF_6/O_2 at 25% O_2 concentration. No enhanced etching was observed indicating the oxide layer effectively inhibited MAPE. The scale bar represents 400 nm.
